# Use of Carbotrace 480 as a Probe for Cellulose and Hydrogel Formation from Defibrillated Microalgae

**DOI:** 10.3390/gels8060383

**Published:** 2022-06-16

**Authors:** Frederik L. Zitzmann, Ewan Ward, Avtar S. Matharu

**Affiliations:** Green Chemistry Centre of Excellence, Department of Chemistry, University of York, York YO10 5DD, UK; ew1057@york.ac.uk

**Keywords:** Carbotrace 480, microalgae, defibrillated celluloses, hydrogels

## Abstract

Carbotrace 480 is a commercially available fluorescent optotracer that specifically binds to cellulose’s glycosidic linkages. Herein, the use of Carbotrace 480 is reported as an analytical tool for linking cellulose content to hydrogel formation capability in defibrillated celluloses obtained from proprietary microalgae. Defibrillated celluloses obtained from acid-free hydrothermal microwave processing at low temperature (160 °C) showed poor hydrogel formation attributed to a low cellulose concentration as evidenced through the lack of Carbotrace fluorescence. High temperature (220 °C) processing afforded reasonable gels commensurate with a higher cellulose loading and stronger response to Carbotrace.

## 1. Introduction

Microalgae are typically unicellular species that grow in aquatic environments using sunlight and carbon dioxide as sources for photosynthesis to generate their energy [1,2,3]. They mainly consist of carbohydrates, lipids, proteins and, pigments, the exact split of which varies from species to species [4]. Their photosynthetic ability, coupled with their abundance across oceans, rivers, and lakes, allows microalgae to fix massive amounts of carbon dioxide from the atmosphere with estimates of around 865 mg CO_2_ L^−1^ day^−1^ [2,5].

Microalgal cell walls comprise a complex three-dimensional structure that varies heavily depending on the species. Generally, microalgal cell walls consist of carbohydrates, lipids, and proteins, with carbohydrates being the most abundant cell-wall building block and proteins only making up around 5–8% of the dry cell-wall mass [6]. There is, however, a lack of scientific research into the exact structural composition, and only very well-known species are well-documented.

Cellulose as a cell-wall component in microalgae plays a lesser role than in terrestrial biomass. In addition to the lesser abundance, the microalgal cell wall is very thick and rigid, making extractions without any pre-treatment very difficult [7,8,9]. Microwave-assisted extraction (MAE) is a very efficient way to disrupt algal cell walls by creating localized pressure waves formed from the dielectric heating of water within the samples and aiding the production of defibrillated celluloses (D.C.) [10,11,12,13]. The microwave process induces hydrolysis of carbohydrates and therefore aids in the weakening and disruption of the cell wall, making extraction easier.

The MAE-derived D.C. can then be further processed to form cellulose-based hydrogels. Cellulose as a gelling agent in combination with water is well-studied [10,11,13]. The abundant hydroxyl groups in cellulose possess the capability to capture and trap water in the wider cellulosic structure through hydrogen bonding to form a three-dimensional hydrogel [14,15,16]. These hydrogels derived from biomass are becoming increasingly sought after in various fields of application, such as hygiene products, contact lenses, wound healing products, or lubricants [15]. Due to cellulose-based hydrogels being both biodegradable as well as low-cost and highly abundant, they pose a very promising and green candidate for future innovations.

Carbotrace molecules are fluorescent optotracers which specifically bind to the glycosidic linkages in cellulose and are therefore able to visually map cellulose content using confocal laser microscopy [17,18,19]. They have previously been used to identify cellulose in plant cells for mapping cellulosic nanofibrils in microfluidic devices and anatomical mapping in plant cells [17,18,19]. However, this paper reports the first use of Carbotrace to analyse defibrillated cellulose obtained from microalgae which were used for the formation of hydrogels.

This paper aims to use Carbotrace 480 for the first time to explore the distribution of cellulose in defibrillated cellulose samples obtained from microalgae prepared according to the method detailed by Zitzmann et al. [10]. The findings of the analysis using Carbotrace 480 will be linked to the hydrogel formation capabilities of the samples. The D.C. samples have been prepared using this acid-free and TEMPO-free green extraction process [10,11,12]. Carbotrace 480 acts as a non-destructive analytical tool for cellulose content in defibrillated cellulosic matter obtained via acid-free microwave hydrothermal processing of microalgae and their propensity to form hydrogels. The Carbotrace 480 (CT 480) was chosen, amongst many trials, to be the ideal probe molecule as its emission maxima did not interfere with the autofluorescence of the defibrillated celluloses. Therefore, the use of CT 480 allows for a good resolution of the images and a clear designation of cellulose and the other parts of the defibrillated celluloses that do not bind to Carbotrace 480.

## 2. Results and Discussion

### 2.1. Formation of Hydrogels from Defibrillated Cellulose Samples

Hydrogels have been formed from defibrillated cellulose samples prepared from both native microalgae and spent microalgae (industrial processing of ethanol-based alkali extraction of lipids leaving spent biomass) as detailed in Zitzmann et al. [10]. Defibrillated cellulose samples have been prepared according to the method previously described [10]. Results of the hydrogel formation are summarized in Table 1.

**Figure 1:**
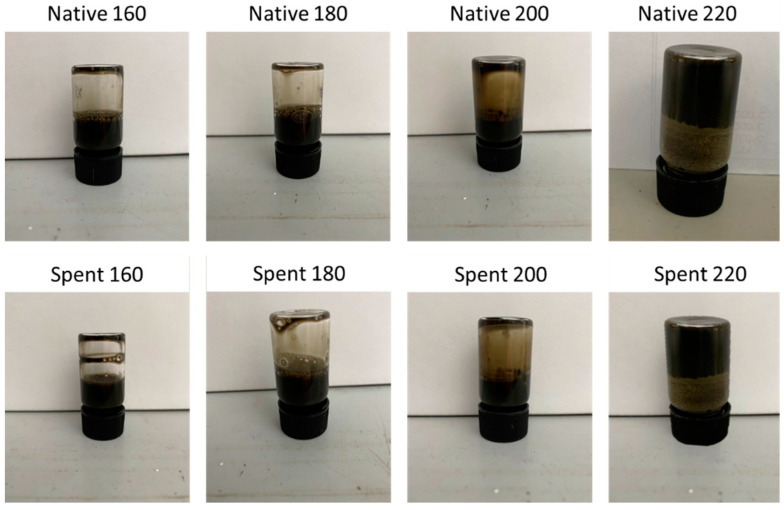
Hydrogels formed from defibrillated cellulose samples obtained from native algal biomass and spent algal biomass. Numbers refer to the temperature in °C of the microwave process used for production of the D.C.

It was only possible to form hydrogels for the defibrillated cellulose samples that have been treated in the microwave at the highest temperature (Figure 1). All other samples did not yield any stable gel, merely a thick viscous mixture which, upon inversion of the vial, did not show any stability. The hydrogel formed from the Native 220 and Spent 220 samples formed a stable gel upon inversion but began to disintegrate within a couple of minutes after preparation.

With conventional analytical methods such as XRD or solid-state NMR, no clear direct correlation between the ability to form hydrogels and the cellulose content could be established, contrary to other literature findings [11,12] due to the increasingly complex mixture of cell-wall components in microalgae compared to plant cells. Therefore, Carbotrace has been used as a probe to identify the cellulose content in the samples and establish a link to the ability to form hydrogels.

### 2.2. Using Carbotrace 480 as a Probe to Image Cellulose Content

**Figure 2:**
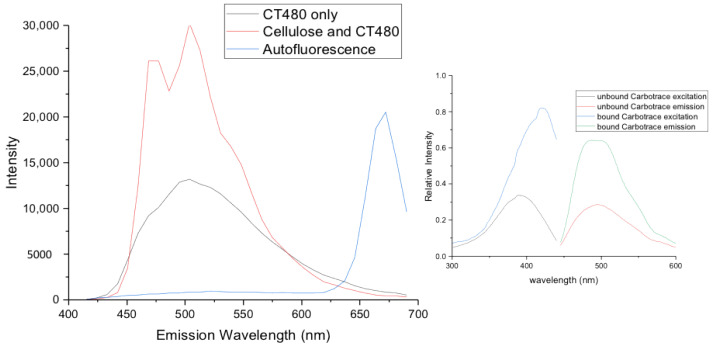
Confocal Laser Microscopy emission spectra of unbound CT480, bound CT480 to pure cellulose, defibrillated cellulose autofluorescence, and their comparison to the reference spectra provided by Ebba biotech—right side.

To generate reference spectra of both the pure cellulose channels and the autofluorescence of the samples to apply to all the later images for unmixing and clear assignment, as well as to replicate the literature excitation and emission spectra of bound and unbound CT480, CT480 was run on its own in phosphate-buffered saline (PBS) as well as bound to pure cellulose immersed in PBS (see Figure 2).

The obtained emission spectra correlate well with the spectra provided by Ebba Biotech with the emission maxima in the region of 480 nm as well as the perceived shift to the left upon Carbotrace binding to cellulose (approx. 20 nm). The intensity in emission also increases by around a factor of two upon binding to cellulose indicating that the intense fluorescence capabilities of the Carbotrace 480 are being switched on due to structural changes in the optotracer backbone upon binding to cellulose.

Also, with the autofluorescence peaking at around 670 nm and no emission appearing in the region where Carbotrace emits, there is excellent spatial separation between the two allowing for confident de-mixing of the channels of the subsequent defibrillated celluloses.

**Figure 3:**
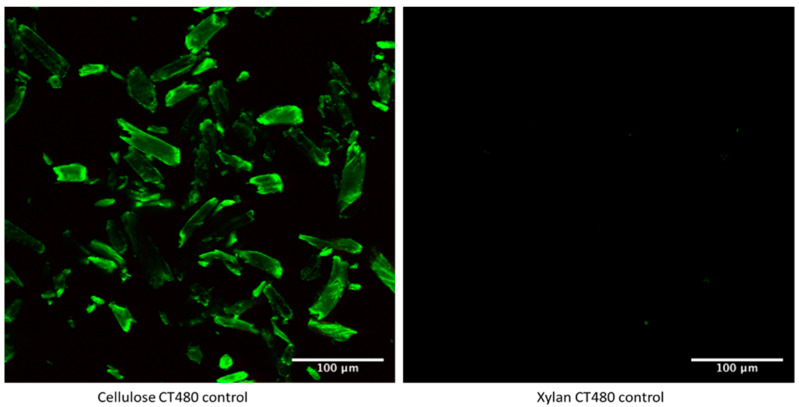
Laser Confocal Microscopy images of CT480 mixed with pure cellulose (**left**) and pure xylan (**right**) with the exact same instrument settings.

In order to confirm that the CT480 binds to cellulose only and not to other carbohydrate structures that can be found in algae, for example, xylan, both cellulose and xylan were mixed in their pure form with CT480. The images in Figure 3 show that, with the exact instrument settings, the pure cellulose manifests a very bright response to the CT binding, whereas xylan stays almost exclusively black (dark), confirming that the Carbotrace indeed binds to cellulose only.

**Figure 4:**
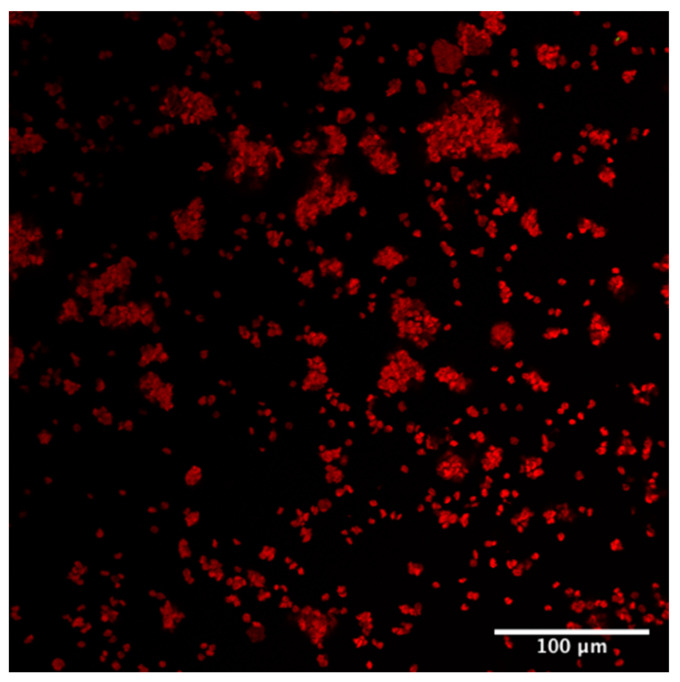
Laser Confocal Microscopy Image of unstained defibrillated cellulose 220 from native biomass.

The morphology and aspect ratio of the D.C.s also clearly reveal themselves. In order to further make sure that the emission reference giving rise to the green colour channel only refers to the CT480 bound to cellulose, the native defibrillated cellulose 220 was run without any Carbotrace (Figure 4) to confirm the autofluorescence as the only component detected.

The pure red colour channel obtained (Figure 4) was used to generate the autofluorescence emission spectrum used as a reference for all later defibrillated celluloses to de-mix cellulose bound to Carbotrace and autofluorescence the rest of the sample.

**Figure 5:**
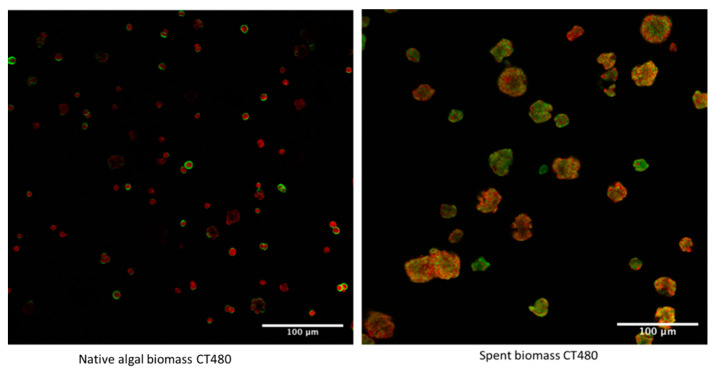
Confocal Laser Microscopy image of initial native and spent biomass mixed with Carbotrace 480.

To explore the initial untreated biomass and the differences in cellulose distribution across the microalgae, Figure 5 shows both the initial native and spent biomass mixed with CT480.

**Figure 6:**
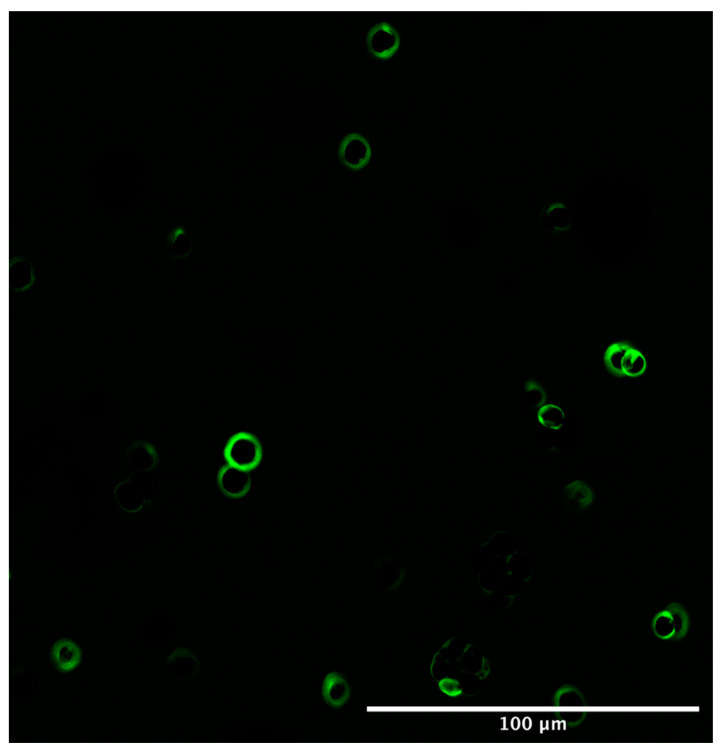
Laser Confocal Microscopy Image of the cellulose-only channel with CT480 of initial native biomass.

The differences between the initial native and spent biomass can be seen very clearly in these images, with the native biomass showing an array of single microalgal cells, each with a ring of cellulose encapsulating the cells. This is in line with the basic structure of microalgal cells which has a cell wall containing cellulose wrapped around the inner cell. This is displayed more clearly in Figure 6, where only the cellulose channel is shown. Furthermore, the Carbotrace binds well to the cells and shows the cell wall.

On the other hand, the spent biomass shows a much more disrupted profile with no individual circular cells able to be made out anymore. Rather an array of smudged and smashed cells with irregular shapes and a more even distribution of cellulose across the whole cell are observed, indicating that the industrial process has indeed destroyed and ruptured the cells. This is in line with previous findings that suggest an easy extraction from spent biomass precisely due to the factors that the image shows of a completely disrupted cell [10].

**Figure 7:**
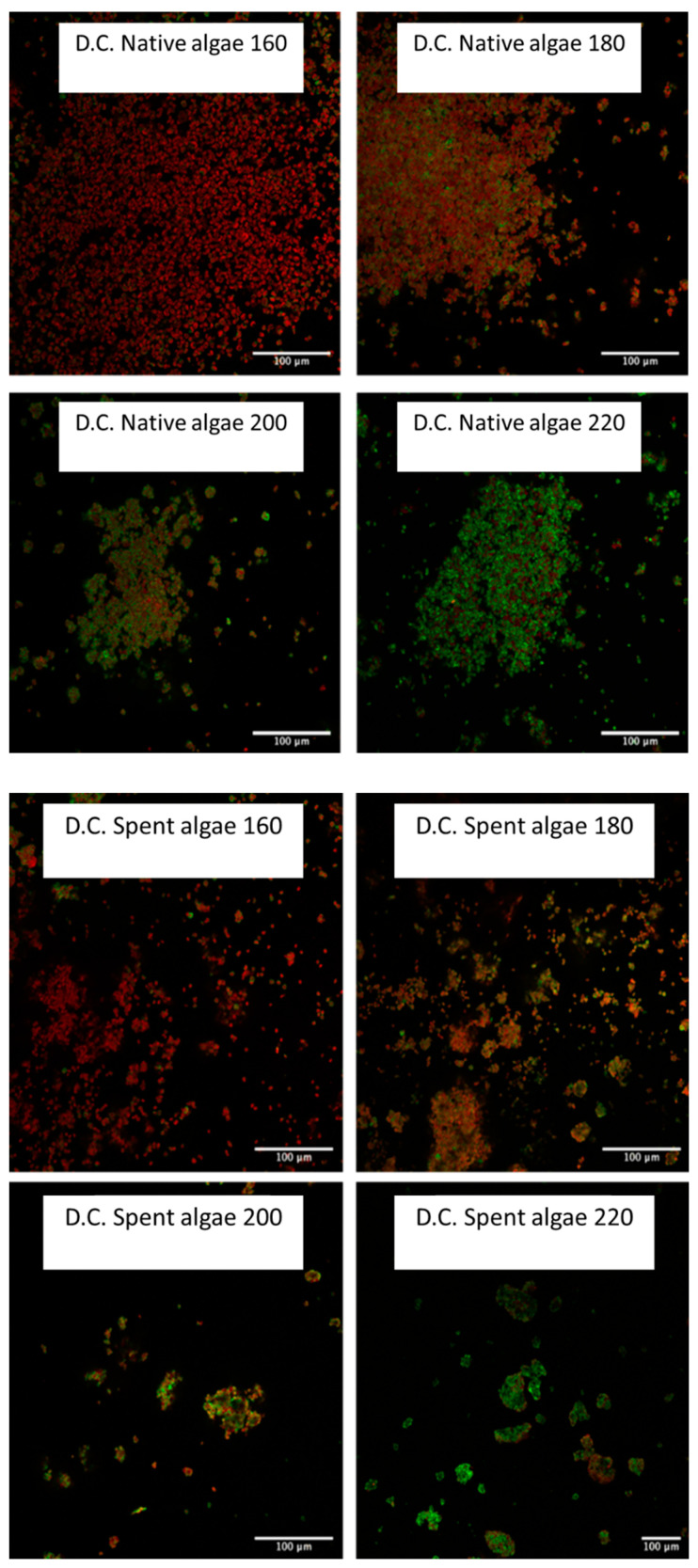
Laser Confocal Microscopy Images of all native algal derived and spent derived defibrillated celluloses.

In order to apply the Carbotrace technology to the defibrillated celluloses and to use a visual tool that can directly identify and spatially show the distribution of the cellulose, all eight defibrillated cellulose samples were mixed with the Carbotrace 480, with the results displayed in Figure 7.

The results show that both types of defibrillated celluloses form very small grains which then lump together into larger aggregates. Both types of defibrillated celluloses clearly show the trend that, with increasing microwave temperature, an increasing amount of cellulose can be observed in the samples, which is in line with previous findings from Zitzmann et al. that suggested that the highest temperatures show the highest correlation with pure cellulose [10].

However, it is interesting that, at the lowest temperature, there is still very little sign of cellulose as the red autofluorescence is much more prominent than any green specks, indicating the presence of cellulose. At the highest temperatures, this ratio is reversed with mostly cellulose being present in the samples. It must be noted that this type of analysis is suitable only for qualitative analysis and cannot be used to quantitatively calculate the exact percentages of cellulose present in the samples. Nevertheless, it still confirms visually what the previous analyses (XRD, NMR) hinted at, namely that cellulose content increases with higher microwave temperatures [10].

**Figure 8:**
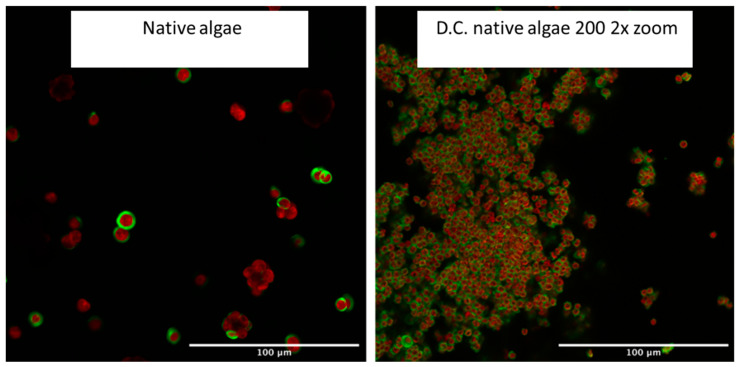
Laser Confocal Microscopy Images of initial native algal biomass and defibrillated celluloses 200 from native biomass at 2× zoom.

Figure 8 shows the nucleus/grain formation of the defibrillated celluloses with the native 200 defibrillated celluloses as a very clear example next to the initial native biomass. The nucleus formation and aggregate formation can be seen very clearly, which bears some resemblance to the initial microalgal cells; however, there is a size difference of 4–5 times that can be made out when comparing them to the initial spray-dried biomass on the left. Also, the shape of the grains is less perfectly circular but rather slightly off-shape. Interestingly, it can be seen that there is again encapsulation of the core by the cellulose which is flagged as green by the Carbotrace. This might be due to the core of the grains being so dense that the Carbotrace molecules are not able to penetrate and therefore form a circular layer around it.

## 3. Conclusions

The reported findings in this paper, which used Carbotrace 480 as an analytical tool to visualize cellulose content in the defibrillated cellulose samples derived from two types of microalgae, correlate very well with the observed capabilities in forming a hydrogel. The unique fluorescent abilities of the Carbotrace 480 provide a clear link between the cellulose content in the samples, which can be used as a proxy for successful hydrogel formation. It has been shown that, for microalgal samples, a very high percentage of cellulose is required to be able to form any kind of even lightly stable hydrogel.

## 4. Materials and Methods

### Source of Biomass

Microalgae were obtained from AlgaeCytes, Kent, England, who provided omega-3 enriched biomass from their proprietary microalgal Eustigmatophyceae strain, ALG01. The ALG01 strain was upscaled from Petri dish to 100 L using AlgaeCytes in-house proprietary upstream pyramid process and inoculated into the 1000 L Industrial Plankton seeding tank. Once the culture reached the late exponential phase, it was transferred into AlgaeCytes pilot-plant production module (VariconAqua 12,000 L Phyco-FlowTM). After reaching an appropriate density, it underwent semi-continuous harvesting to provide material for spray drying. On each harvesting day, 1000 L of algal culture was dewatered using an Alfa Laval Clara 20 model disc-stack centrifuge to produce an algal slurry of ~15% ± 5% solids. The algal slurry was subsequently dried using a Büchi mini spray dryer B-290 to produce a dried algal powder of <1% moisture content.

The defibrillated cellulose samples were prepared according to the method detailed by Zitzmann et al. [10] and using a Milestone Synthwave reactor (1500 W, 2.45 GHz).

Hydrogels were produced by mixing the defibrillated celluloses with deionized water (3 wt%) and treating the mixture with a homogenizer for 3 min. The stability of the gel was tested by inverting the vial and recording the time it stably stayed at the top of the vial before descending.

Carbotrace 480 was obtained from Ebba Biotech (Stockholm, Sweden). It was mixed with PBS at a ratio of 1:1000 (pH 7.4) and, subsequently, the defibrillated cellulose samples (0.2 mg) were mixed with aliquots of this stock solution (50 µL) and left to incubate for 30 min at room temperature.

Carbotrace images were captured using a Zeiss LSM980 confocal microscope, AxioObserver Z1 using ZEN 3.4 (blue edition) software and either an EC Plan-Neofluar 10×/0.3 or a Plan Apochromat 20×/0.8 objective. All samples were excited with a 405 nm laser using a 405 nm main beam splitter, and emissions were collected from 411–694 nm in bins of 8.9 nm. The pixel size was 1.657 μm^2^ or 0.829 um^2^ for the 10× or 20× objectives, respectively. The pinhole was 1 AU, and the images were taken in 16 bit.

Reference spectra of cellulose stained with Carbotrace 480 and autofluorescence values from unstained sample spectra were collected independently to permit optimal spectral unmixing. Samples were typically averaged ×8 to reduce noise and increase the precision of the spectral unmixing. This process was performed using the in-built application within the Carl Zeiss ZEN 3.4 software (Jena, Germany) on a pixel-to-pixel basis.

The images were unmixed as follows:

SY_temperature samples using SY160 unstained autofluorescence spectra and the cellulose CT480 spectra.

ST_temperature samples using ST160 unstained autofluorescence spectra and the cellulose CT480 spectra.

## Figures and Tables

**Table 1:** Hydrogel formation results of defibrillated cellulose samples with numbers corresponding to the temperature in °C of the microwave process in the preparation step of the samples.

Sample Type	Hydrogel Formation
Native 160	X
Native 180	X
Native 200	X
Native 220	Reasonably stable gel
Spent 160	X
Spent 180	X
Spent 200	X
Spent 220	Reasonably stable gel

## Data Availability

Not applicable.
